# Bringing machine learning to research on intellectual and developmental disabilities: taking inspiration from neurological diseases

**DOI:** 10.1186/s11689-022-09438-w

**Published:** 2022-05-02

**Authors:** Chirag Gupta, Pramod Chandrashekar, Ting Jin, Chenfeng He, Saniya Khullar, Qiang Chang, Daifeng Wang

**Affiliations:** 1 Waisman Center, University of Wisconsin-Madison, Madison, WI 53705 USA; 2 Department of Biostatistics and Medical Informatics, University of Wisconsin-Madison, Madison, WI 53706 USA; 3 Department of Medical Genetics, School of Medicine and Public Health, University of Wisconsin-Madison, Madison, WI 53705 USA; 4 Department of Neurology, School of Medicine and Public Health, University of Wisconsin-Madison, Madison, WI 53705 USA; 5 Department of Computer Sciences, University of Wisconsin-Madison, Madison, WI 53706 USA

**Keywords:** Intellectual and developmental disabilities, Machine learning, Artificial intelligence, Genomics, Multi-omics, Brain

## Abstract

Intellectual and Developmental Disabilities (IDDs), such as Down syndrome, Fragile X syndrome, Rett syndrome, and autism spectrum disorder, usually manifest at birth or early childhood. IDDs are characterized by significant impairment in intellectual and adaptive functioning, and both genetic and environmental factors underpin IDD biology. Molecular and genetic stratification of IDDs remain challenging mainly due to overlapping factors and comorbidity. Advances in high throughput sequencing, imaging, and tools to record behavioral data at scale have greatly enhanced our understanding of the molecular, cellular, structural, and environmental basis of some IDDs. Fueled by the “big data” revolution, artificial intelligence (AI) and machine learning (ML) technologies have brought a whole new paradigm shift in computational biology. Evidently, the ML-driven approach to clinical diagnoses has the potential to augment classical methods that use symptoms and external observations, hoping to push the personalized treatment plan forward. Therefore, integrative analyses and applications of ML technology have a direct bearing on discoveries in IDDs. The application of ML to IDDs can potentially improve screening and early diagnosis, advance our understanding of the complexity of comorbidity, and accelerate the identification of biomarkers for clinical research and drug development. For more than five decades, the IDDRC network has supported a nexus of investigators at centers across the USA, all striving to understand the interplay between various factors underlying IDDs. In this review, we introduced fast-increasing multi-modal data types, highlighted example studies that employed ML technologies to illuminate factors and biological mechanisms underlying IDDs, as well as recent advances in ML technologies and their applications to IDDs and other neurological diseases. We discussed various molecular, clinical, and environmental data collection modes, including genetic, imaging, phenotypical, and behavioral data types, along with multiple repositories that store and share such data. Furthermore, we outlined some fundamental concepts of machine learning algorithms and presented our opinion on specific gaps that will need to be filled to accomplish, for example, reliable implementation of ML-based diagnosis technology in IDD clinics. We anticipate that this review will guide researchers to formulate AI and ML-based approaches to investigate IDDs and related conditions.

## Introduction

Intellectual and developmental disabilities (IDDs) usually begin at birth (but can manifest anytime during a child’s developmental trajectory before the age of 18). IDDs can limit the functioning of an individual’s nervous, sensory, or metabolic function and may be potentially degenerative over time [1]. Intellectual disability (ID) is a condition characterized by below-average cognitive abilities. Individuals with IDs often have impaired learning, language, behavior, and social skills. Individuals with developmental disabilities (DD) have severe chronic often lifelong disabilities that can be intellectual, physical, or both. The term DD may encompass IDs, but individuals with DD may not always exhibit impaired cognitive abilities (e.g., in blindness). IDD is a broad term used to describe situations where ID and DD are present. For example, cerebral palsy, Down syndrome (DS), fragile X syndrome (FXS), and autism spectrum disorders (ASDs) can limit intelligence and cognitive abilities by affecting the central nervous system. Adults with IDDs are more prone to sensory impairment (hearing and visual) than the general population [2, 3]. Studies on these conditions have identified various genetic causes and implicated environmental factors, such as prenatal exposure to hazardous chemicals or radiation. However, the phenotypic boundaries between different IDDs are not always very clear.

Technological innovations in sequencing and imaging have put many areas in medicine on the brink of data-driven transformation. Our ability to share and access biological data generated in different laboratories is growing exponentially, fueling secondary analysis and data reuse by independent researchers [4]. With this surge in the volume of data in centralized repositories, it is not uncommon to have access to multimodal data that can be integrated and turned into actionable insights. Research on IDDs, and neurological diseases in general, has also witnessed this “big data” revolution, although slower than other diseases and disorders. The National Institute of Mental Health has shifted focus from clinical research and trials to data-driven understanding of the biological mechanisms and causal models of mental illnesses. The wide range of modalities and data types spanning neurological diseases provides an exciting opportunity to develop computational models based on artificial intelligence (AI) and machine learning (ML) techniques, facilitating translational research, therapeutic decision-making, and patient care.

The central tenet of AI and ML techniques is to computationally automate logical reasoning by extracting general rules and patterns from large datasets. An ML model is essentially a mathematical function that takes input data, generalizes it well, and maps it to an outcome with high levels of accuracy. ML techniques have been applied in various sciences where a large amount of data can be acquired in a high throughput manner from multiple streams. With the open-access mindset of the scientific community and the rise of centralized storage databases, ML and AI naturally found applications in the field of genetic medicine and healthcare.

In neuroscience, the most prominent data type is imaging. Neuroimaging broadly involves non-invasive techniques that allow scientists and clinicians to study the anatomy of the human brain in vivo (medical experiments or tests performed on living organisms). Multiple modalities in neuroimaging traditionally refer to various imaging tools used to collect data from a subject. Imaging modalities allow the interrogation of critical parameters related to the brain’s structure, function, and pharmacology. For example, imaging techniques such as X-ray CT scans provide structural maps of the brain. Positron emission tomography (PET) and magnetic resonance imaging (MRI) techniques, invented relatively recently, correlate the structural representation with functional activities [5]. The maps of localized brain activities make it possible to make deductions on the correlation between brain structure and function.

The past few years have also witnessed rapid advances in the generation of omics data across multiple modalities. It is now relatively routine to study health and disease using genomic biomarkers, such as single-nucleotide variants (SNVs), copy number variants (CNVs), insertions, deletions, and other DNA-level anomalies. Moreover, the next-generation sequencing (NGS) technology allows us to interrogate cellular functions at various levels. For example, transcriptomics quantifies RNA molecules in the cells, which can shed light on gene activity dynamics in varying conditions. Epigenomics assays measure which DNA regions are accessible to the transcriptional machinery, e.g., transcription factors (TFs) binding and histone modifications. While proteomics quantifies the protein composition of the cells, metabolomics instead quantifies various metabolites that accumulate in the cells. Thus, integrated analyses of these omics data types offer a holistic understanding of the biosystem. However, the most preferred data type in omics are genomics and transcriptomics mainly because they are relatively inexpensive to obtain and straightforward to analyze, offering a genome-wide assay of the system.

Apart from genomics and imaging data, other data streams such as digitized historical health records, voice and motion features, and familial and environmental data can also be helpful. Big data analytics, spanning all brain-related disorders, might help quantify biological and clinical differences between disorders with overlapping genetic liability and clinical symptoms. Emerging techniques in ML could empower doctors and clinical practitioners in diagnosis and drug developers in prioritizing lead candidates.

**Fig. 1:**
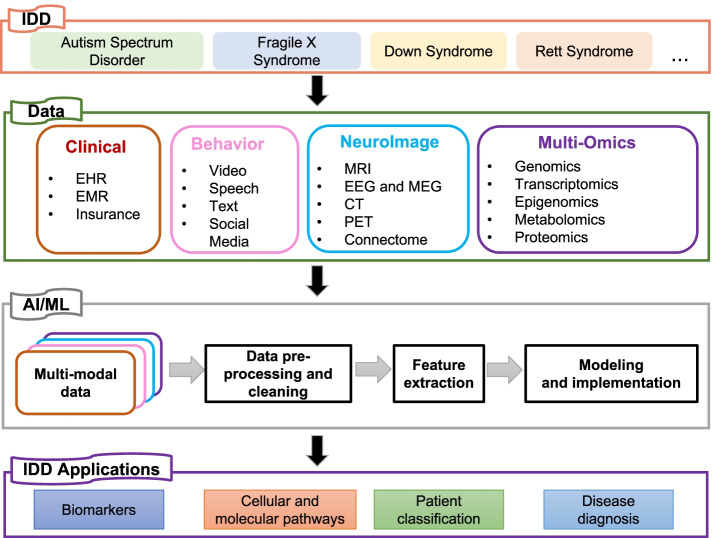
An overview of the ML process and potential applications to IDDs. Various types of data (e.g., clinical, behavior, neuroimaging, and multi-omics) are usually recorded in IDD cohorts. These data sets are first individually processed and cleaned to remove noise and extract relevant biological signals (feature extraction). Then, an AI/ML algorithm is trained to find rules and patterns in the integrated dataset. The choice of the algorithm usually depends on the formulation of the biological problem and other data-set specific factors (discussed in Machine learning methods and applications to IDDs section of the main text). Typically, the model can be tested objectively with independent datasets or prior knowledge. A correctly evaluated and validated model is often generalizable, and such models have a variety of clinical and laboratory applications in IDDs

As summarized in Fig. 1, capitalizing on the available multimodal datasets can unlock new possibilities for discoveries and applications in IDDs. In this review, we discussed the various genetic, imaging, and clinical technologies used as modes of data acquisition and the potential applications of these multimodal data for understanding multiple conditions associated with IDDs. We explained the underlying biology and explored the current evidence regarding genetics, genomics, and multimodal imaging data, and some example studies that applied ML models and AI implementations to identify factors underlying IDDs and related disorders. Furthermore, we also reviewed some fundamental technical concepts in AI and ML along with current challenges in leveraging these approaches to understand IDD biology. Finally, we outlined general-purpose, open-source tools developed by computational neuroscientists.

To find relevant articles on a given topic (e.g., GWAS studies in autism, ML in fragile X syndrome, etc.), we made advanced PubMed searches by pairing two key terms using the “add with AND” in the query box (e.g., autism AND GWAS, machine learning AND fragile X syndrome, etc.). Given the vast scope of our review, covering multiple modalities in various IDDs, we included those articles that (a) applied NGS, imaging, or other high throughput technologies to study IDDs; (b) applied an ML framework to IDD datasets; and (c) in cases where no relevant IDD study could be found on an important concept, we used example studies from other neurological diseases (e.g., for multi-modal data integration). In some cases, we also included other review and opinion articles that discuss fundamental concepts on a given topic (e.g., general limitations of ML). Based on these criteria, of all the articles, we included in this review, 40.2% involve a disorder classified as IDD (ASD, FXS, DS, ADHD, and cerebral palsy), 3.8% involve an IDD in conjunction with other neurological or neuropsychiatric disease, 40.2% involve neurological or neuropsychiatric disease exclusively (without IDDs), and the remaining articles are ML protocol or other review papers within this context.

Based on these articles, we categorized reviewed works in the following manner. In Multimodal data for IDDs and neurological diseases section, we first reviewed various data types that are typically generated or can be easily generated for IDD research. This includes brain imaging, NGS, the use of organoids, and experimental models, behavioral data, and electronic health records. In Machine learning methods and applications to IDDs section, we review ML approaches that are applied to such data types. In this section, we describe applications of ML to single modal data as well as integration of multi-modal data. In Available AI and ML implementations section, we reviewed how advanced ML systems are currently being applied to IDD research. Throughout, we also draw examples from studies aimed at other brain diseases that may not fall under the purview of IDD. Finally, in Conclusions and future directions section, we present our opinion on the current limitations ML and AI frameworks to study IDDs, and how some of the issues can addressed in the future.

### Multimodal data for IDDs and neurological diseases

#### Neuroimaging

Neuroimaging has played an essential role in understanding the connection between brain structure and function and how they relate to various disorders. Neuroimaging can be classified into two types: structural imaging and functional imaging. While structural imaging mainly deals with the brain structure and is used to diagnose injuries or tumors, functional imaging deals with brain activity. Thus, neuroimaging helps in characterizing neurodevelopmental and neurodegenerative diseases. Widely used neuroimaging methods include MRI (both structural (sMRI) and functional (fMRI)) and electro- and magneto-encephalography (EEG and MEG, respectively). MRI is a non-invasive imaging technique that uses magnetic and radio frequencies to create images of brain structure to study anatomy. fMRI scans the brain’s neural activity changes through a series of MRIs, providing good spatial resolution but poor temporal resolution.

EEG records brain activities and changes in the activities using electrodes attached to the scalp. They provide a direct measure of neural activity with in-depth temporal resolution. MEG records the magnetic activities to offer a very high temporal and spatial resolution. EEG and MEG provide complementary information and are often recorded together [6]. Various analyses on the resting state EEG have identified early disruptions in brain oscillations [7–9], weaker functional connectivity in the frontal lobe [10, 11], and mirror neuron system dysfunction among ASD individuals [12, 13]. Auditory processing abnormalities have also been detected in FXS through EEG images [14].

CT scans are radiological images that use X-rays to scan the body to obtain cross-sectional images. PET uses radioactive tracers to detect metabolic activities, blood flow, and neurotransmitters and provides images of the cellular function in various tissues. While CT scans and MRI can detect tissue level anomalies, PET scans detect anomalies at a cellular and metabolic level [15]. For example, PET scans have shown an increased serotonin synthesis capacity in autistic children [16].

The rapidly evolving imaging technologies provide rich information sources that capture different information about human subjects. When combined, these datasets can potentially provide a better perspective on the subject’s features. Several multimodal imaging datasets are publicly available for such integrative analyses. The LUMED dataset [17] consists of EEGs and facial expression images of 13 participants (6 females and 7 males) in the EEG paradigm. DEAP [18] contains the EEG and physiological signals of 32 participants who were made to watch 40-min-long music videos. The multimodal SEED dataset [19, 20] contains EEG signals of 23 subjects (12 females, 11 males) who were asked to participate in the virtual reality-based driving system, where they drove a car in various simulated scenarios without alertness. Although most of these mentioned EEG datasets are not disease-specific yet, they provide a perspective on normal human behavior, affective states, and emotions, which can be utilized to find anomalies in larger disease cohorts.

#### Next-generation sequencing data

##### Transcriptomics

Transcriptome analysis measures the level of gene expression in individual cells or tissues. Gene expression analysis can provide helpful information about the dynamics of cellular states across multiple conditions and developmental stages. Typically, gene expression analysis involves identifying differentially expressed genes in healthy and disease conditions and investigating perturbed pathways and cellular processes represented by those genes. In IDDs, transcriptomic studies show consistent gene expression patterns involved in brain development and neuronal activity [21]. These observations hint at underlying convergent molecular pathways involved in the diseases. Other studies comparing the transcriptomes of disease groups versus healthy groups have also identified several genes that differentially express and the underlying pathways [22–26].

However, because a single study can be limited by the sample size and confounding factors such as sample sources and the experiment bias, meta-studies have been conducted by gathering transcriptome data from free public resources [27–30]. Meta-analysis is a statistical analysis that combines results from multiple separate studies focused on answering the same question. For example, Jaume et al. performed a meta-analysis with samples from two studies and identified 1567 differentially expressed genes (DEGs) in the cortex of ASD patients [27]. Carolyn et al. performed a meta-analysis of over 1000 microarrays from 12 independent studies of healthy individuals and ASD patients [28]. Their study identified several known/novel DEGs indicating a typical transcriptomic signature across multiple independent groups of individuals with ASD. Some meta-analysis studies have also found commonalities between IDD and other human diseases, e.g., ASD with cancer [30]. The Gene Expression Omnibus [31], ArrayExpress [32], and dbGaP [33] are excellent open repositories of large-scale transcriptome datasets on which meta-analysis can be performed.

##### Epigenomics

The epigenome is a multitude of chemical modifications that direct genome function by activating or repressing specific genes and thus affecting the state of the transcriptome. Epigenetic mechanisms lie at the interface between the genome and the environment [34] (nature versus nurture), which alter gene expression without changing the underlying DNA sequences. These epigenetic changes are reversible responses to environment and behaviors (e.g., diet, exercise, up-bringing, aging, stress, other lifestyle choices) and typically may involve DNA modifications (adding a chemical group to DNA that may turn on or off a gene), histone modifications (change whether a gene region of the DNA is wrapped tightly around a histone protein and is inaccessible for transcription, switched “off”), and non-coding RNA (may recruit proteins to modify histones that help control accessibility of the gene). Since IDDs are highly affected by the environment, studying epigenomic data points may help us understand the causality of disorders and possibly its progression.

Recent advances in epigenomics technologies allow profiling higher-order chromatin folding structures (e.g., Hi-C, chromatin accessibility, and DNA methylation) [35–37]. These techniques have been widely applied to study various IDDs [38]. For example, Nardone et al. measured the methylation status of two cortical regions of ASD patients and identified several epigenetic changes and biological processes within the synaptic and immune categories [39]. DNA methylation analysis of autistic brains reveal multiple dysregulated biological pathways. A case-control meta-analysis of DNA methylation from 968 blood samples of ASD children identified 55 ASD-associated methylated CpG sites [40]. Nardone et al. also identified more than 10,000 differentially methylated CpG sites in two cortical regions of individuals who had ASD [39].

##### Single-cell omics

One trend that is recently surging in the genomics domain is a single-cell sequencing technology. Sequencing at the single-cell level allows researchers to study the cellular heterogeneity of the brain by profiling tens of thousands of individual cells [41, 42]. Understanding how gene functionality and expression differs for different cell types in the brain will be invaluable as these cell types play key roles in various brain diseases. For instance, a study found PAK3 mutations in mental illnesses with intellectual disability and found PAK3 is strongly expressed in oligodendrocytes and precursors, suggesting that depression, ASD, and SCZ may involve oligodendrocytes (a glial cell that mainly produces and maintains myelin sheath to insulate neuron axons) [43]. Hence, single-cell technologies can help uncover more disease genes and non-coding variants at a cellular level since gene expression and regulation can differ based on cell-types and be specific to brain diseases.

While single-cell sequencing technologies have been extensively applied to study the cell types of the human brain [44–46] and their activity in Alzheimer’s [47], relatively fewer studies have applied single-cell sequencing to study IDDs. For example, Dmitry et al. measured the single-cell transcriptome of cortical cells of 15 ASD patients and controls and identified cell types preferentially affected in ASD patients [48]. In addition, Nagi et al. sequenced ~80,000 cells from the prefrontal cortex of 17 individuals with major depressive disorder and healthy controls [49]. Their study showed that 47% of the observed gene expression changes were likely caused by dysregulation of excitatory neurons and immature oligodendrocyte precursor cells [49].

##### Genetic variants and genome-wide association studies

The advent of high-throughput sequencing technologies and advanced bioinformatics tools made it possible to link human diseases to DNA sequence anomalies. Specific genetic changes, such as single-nucleotide variants (SNVs), copy number variants (CNVs), and other large structural variations (SVs), have been linked to many IDD-related disorders. For example, in non-syndromic ID, 55% of reported variants were found on the X chromosome [50]. DS is linked to an extra copy of chromosome 21. FXS is linked to a CGG expansion in the 5′ UTR of the *FMR1* gene on the X chromosome. However, such a specific genetic change has not yet been identified in all IDDs, mainly due to the complex interplay between genetic and environmental factors (prenatal and postnatal) [51]. Furthermore, IDDs may involve changes in multiple genes, each conferring a small risk, and hundreds of autism risk genes have been cataloged through genomic assays [52–55]. For example, Sanders et al. sequenced the exomes (coding regions of the genome) of 238 families with an autistic child from the Simon simplex collection and found a de novo mutation (DNM; mutations acquired by offspring of healthy parents with no familial history) disrupting three genes (*SCN2A*, *KATNAL2*, and *CHD8*) along with additional risk genes [56]. Later, the team worked on the sequenced exomes from a much larger subset (more than 2500 simplex families in SSC) and found 27 recurrent genes with a 90% chance of being related to ASD [54]. Studies have also found large overlaps between structural variants implicated in multiple disorders. For instance, deletion on chromosomal region 15q13.3 has been linked to intellectual disability, schizophrenia, autism, and epilepsy [57–60]. Similarly, region 16p11.2 has been associated with severe developmental delay, intellectual disability, obesity, schizophrenia, and autism [61–63]. Shared risk CNVs between mental disorders have also been reported. For example, Kushima et al. compared CNVs in ASD and SCZ cases and found 29 pathogenic CNVs common to both disorders [64]. ADHD, ASD, and Schizophrenia also share potentially pathogenic CNVs [65].

With phenotypic and genotypic information collected over large cohorts, the past decade witnessed an exponential surge in genome-wide association studies (GWAS). GWAS is a systematic genome-wide survey of relationships between common sequence variation and disease phenotype, powered by large cohorts of cases and controls. GWAS has been successful in enhancing our understanding of the genetic architecture of neurological diseases such as Alzheimer’s [66, 67], Parkinson’s [68], and epilepsy [69]. However, the relative lack of interest and practical issues such as consent has somewhat hampered the growth of an extensive collection of well-characterized cohorts of individuals with IDDs. This is evident in the GWAS catalog, in which, at the time of this writing, there is only one publication linked with FXS and DS but 24 publications linked with autism. In addition, there are no GWAS studies for cerebral palsy yet. This bias toward studying autism over other IDD conditions could be because individuals with IDD are frequently co-diagnosed with ASD [70] and using ASD as the phenotype in GWAS perhaps provides better power. Nevertheless, there are efforts toward filling these gaps. For example, The London Down Syndrome Consortium (LonDownS) focuses on creating a DS biobank to facilitate more accurately resolved phenotypes for DS GWAS [71].

### Omics data from emerging experimental models

Constructing the spatiotemporal transcriptome/epigenomics landscape changes of healthy human brain development is another approach for understanding disease-induced abnormalities. For example, BrainSpan [72], a consortium across multiple institutions, gathered >1000 samples from 48 postmortem human brains, ranging from prenatal to adult age groups, and measured the transcriptome and epigenome. PsychEncode [73, 74] is another multi-institution consortium that measures multidimensional omics data of approximately 1000 postmortem brains and focuses on understanding gene regulatory mechanisms during human brain development. Encyclopedia of DNA Elements (ENCODE) recently launched an atlas of chromatin accessibility in developing mouse fetuses, of which 1128 ChIP-seq assays and 132 ATAC-seq assays have been performed for 72 distinct tissue stages [75]. Such consortiums, along with the Roadmap Epigenomics Project, provide valuable public resources for researchers to decipher the functional developmental genomics of the mammalian brain. Besides the examples mentioned above, other similar studies have also generated valuable resources for brain research. However, these datasets span multiple institutions or databases, which impedes their use. To overcome this, people have started centralizing data sharing and facilitating other researchers to build upon the existing knowledge. For example, STAB is a collection of transcriptome data from publicly available datasets [76].

Measurements based on postmortem brain samples serve only as snapshots of brain development at different stages, while the precise development of brains is a dynamic process that requires crosstalk among various gene programs, cells, brain regions, and environment, which eventually develop into the brain structure with complex functions. In light of this, brain culture technologies that culture stem cells to differentiate into brain cells have emerged as models of early human development [77]. Traditional in vitro (medical experiments performed in a laboratory dish or test tube) 2D technologies culture induced pluripotent stem cells (iPSCs) in a flat system, which does not fully recapitulate the developmental processes observed during in vivo brain development [78]. This is due to the lack of *z*-axis-related cell-cell interactions in 2D culture systems [79]. To better mimic the biomechanical microenvironment in vivo, 3D brain culture technologies have been developed. These technologies utilize iPSCs to differentiate into brain cells in a 3D structure (organoids) [77]. These technologies have provided unique opportunities and great insights into studying early brain development.

As part of PsychENCODE, Amiri et al. measured the transcriptome and epigenome landscape of 30 organoids [73] and compared those with mid-fetal brain measurements. The authors validated organoids as a suitable model for studying gene regulations in the early stages of human brain development. They found that organoids may help understand the mechanisms of de novo noncoding mutations that are enriched for ASD [73]. Gordon et al. cultured organoids for extended periods (up to 694 days) and measured the transcriptome, epigenome, and RNA editing [80]. The authors observed that the organoids reach a stage similar to the in vivo postnatal stage at 250–300 days, suggesting that organoids can serve as models even at mid- and late-fetal stages [80]. Trevino et al. performed ATAC-seq to measure the chromatin accessibility of organoids cells from long-term culturing (over 20 months) and found the in vitro organoids intrinsically underwent chromatin transitions of in vivo brain development [81]. Kanton et al. compared human organoid to chimpanzee organoid cells at multiple different stages using dynamic time warping to align the pseudo time inferred from single-cell transcriptomic data [82]. The authors observed a similarly delayed maturation of human brains (compared with a chimpanzee) within organoids with what was previously discovered with primary brain samples [83, 84]. A recent landmark study used canonical correlation analysis [85] to compare primary human tissues versus human organoids using a co-cluster of the mixture of cells from both origins [86]. The authors found that the organoids maintained the composition of cell types but varied in the cell percentages. Their findings suggest that using an organoid as a brain model is promising but needs future improvements. Overall, using organoid culturing as models of in vivo human brain development is promising. However, until now, how well the in vitro cultured 3D organoid can mimic the in vivo complex dynamic process remains a question.

Besides culturing iPSC from healthy donors, studies focused on culturing iPSC from IDD patients provided unique opportunities for studying relevant cell types and performing drug testing. For instance, Mariani et al. cultured iPSC-derived telencephalic organoids and found inhibitory neurons are overproduced in organoids from ASD patients [87]. Many studies have focused on this field, and important discoveries have been made with disorder-specific iPSC models. A recent review by Villa et al. details the utility of iPSCs in developing novel therapeutic strategies [88].

### Behavioral data

Behavioral symptoms in social interaction, sensory domain, and motor movements are vital characteristics that are helpful for the assessment and diagnosis of brain-related disorders. With advancements in technology, cameras, sensors, virtual reality, and diverse social media platforms could be used to collect, group, and analyze behaviors.

Visual observation and analysis of natural behaviors have been shown to help with detection of developmental disorders. Several studies discovered and identified early behavioral risk markers of ASD with the help of retrospective analysis of family home videos [89–91]. Baranek et al. analyzed videos to study the early sensory-motor features in infants at 9–12 months with FXS [92]. A few studies focused on extracting the facial expressions in a photo or a video frame, then recognized and labeled the unobtrusive emotions for children with ASD [93, 94]. Movement patterns could also be a significant character for IDD diagnosis. For example, a recent study studied 24 children with ASD and 25 children with typical neurodevelopment who participated in a multimodal virtual reality experience [95]. The researchers tracked changes in the children’s body movements by a depth sensor camera during the presentation of visual, auditive, and olfactive stimuli [95]. They identified the body movements and behaviors that support the assessment of ASD patients [95].

In recent years, data obtained from online forums has also served as a source of behavioral data to detect abnormal behaviors. For example, Mazurek and Wenstrup recorded the amount and intensity of television, video game, and social media usage among children with ASD compared to neuro-typically developing siblings [96]. Saha and Agarwal [97] collected data from an online community and analyzed the interactions among families with autistic individuals. The authors claim to have built a research-based understanding of conversations in social media among families dealing with autism. If done with informed consent and proper regulations, such data sources can help improve diagnosis.

### Electronic health records

The Health Information Technology for Economic and Clinical Health (HITECH) Act of 2009 authorized widespread use of electronic health records (EHRs) [98]. The primary purpose of EHR is to transform the health care system from mostly paper-based records to an electronic-based one. With the increasing use of electronic medical datasets (e.g., EHR data, electronic medical records (EMRs) data, insurance claim records, and other available clinical data), medical providers hope to deliver a superior quality of care to their patients. Such healthcare data could also aid researchers in solving clinical problems and improve clinical outcomes and diagnosis. Several studies used EMR or EHR to examine co-occurring conditions in ASD [99, 100]. For instance, Alexeeff et al. [101] used the medical conditions in EMR to predict whether children in the first year of life would have ASD before they were actually diagnosed and also demonstrated ASD risk stratification. Croen et al. [102] included a large and diverse population of adults aged 18+ with ASD to elevate the co-occurring psychiatric conditions. EHRs from more than one million individuals have also been used to investigate the health characteristics and medical conditions of patients who have been clinically diagnosed with FXS [103].

## Machine learning methods and applications to IDDs

State-of-the-art techniques in ML are now well-positioned to decipher human disease mechanisms with the overwhelming amount of big data. There is a massive influx of accumulating high-dimensional data generated from various genetic, imaging, and clinical technologies, as described above. Greener et al. recently presented an excellent “guide to machine learning for biologists” in which they provided a visual depiction of fundamental concepts in ML and techniques that can be applied to different types of biological data [104]. Various machine learning approaches have already been developed and adopted in computational biology, primarily for data integration, pattern finding, and predictive analysis tasks. Some of the key terminology and concepts used by the ML community are outlined in Table 1.

**Table 1:** Glossary of key terms in artificial intelligence and machine learning

**Feature selection:** Feature selection is a type of dimensionality reduction that involves selecting a subset of features from the original feature set, which can potentially improve a model’s performance. As every feature added to the machine learning model increases the complexity of the model and risk of overfitting (when the model performs well on training data but fails on new data), thereby complicating the inferences. Feature selection aims at reducing redundancy while selecting the most relevant features.	
**Training:** Training a model involves passing the processed data to a machine learning algorithm to learn general rules and patterns in the data. Usually, the goal is to optimize model parameters such that it is generalizable (able to perform well on unseen testing data) while maintaining accuracy.	
**Supervised learning:** Supervised learning is a group of machine learning techniques that use labeled data in the form of prior knowledge (gold standard) as input to train the model. The model learns patterns that characterize samples with known labels, and these patterns can then be used to predict the labels of new data. Regression (continuous value prediction) and classification (discrete value prediction) are two types of supervised learning.	
**Unsupervised learning:** Unsupervised learning is a branch of machine learning that involves training a model using unlabeled data (input without a labeled output) based on structure and intuition. Clustering is a popular example of unsupervised learning.	
**Performance metrics:** Performance metrics help us to assess the performance of the machine learning/deep learning models. Some of the common metrics are as follows:	
a. Confusion matrix: False positives (FP) are the number of negative samples which were wrongly predicted as being positive; false negatives (FN) are the number of positive samples which were wrongly predicted as being negative. Accurate predictions are true positives (TP: number of truly positive samples correctly predicted) and true negatives (TN: number of truly negative samples correctly predicted).	
b. Accuracy (ACC)—This is mostly used for classification tasks. It tells us the ratio of correctly predicted labels among all the labels. It ranges between 0 and 1 where 1 means all samples are correctly predicted and 0 means random guess.	
c. Area under the curve (AUC)—Also used in classification tasks. It tells us how well the model can differentiate among classes at various thresholds. Higher AUCs correspond to models that can better distinguish between disease (usually class 1) and healthy (usually class 0) patients. The values range from 0 to 1 and are usually compared with random guessing (AUC of 0.5).	
d. Mean squared error (MSE)—It is mostly used in regression purposes. It measures the average of the squared difference between the predicted values and the respective ground truth values. Intuitively, it computes the variance of the residuals.	
e. Mean absolute error (MAE)—Widely used in regression tasks, it measures the absolute distance between the predicted and the ground truth labels.	
f. Purity—This metric is used in clustering unsupervised learning approaches. It measures how well each cluster contains an individual class.	
g. F1 score (F1)—Harmonic mean between precision and recall. The values can be between 0 and 1, where predictive models try to achieve F1 scores close to 1.	
**Model evaluation:** Model evaluation involves assessing the generalizability of the model. It helps in determining if the trained model will generalize to unseen data. A popular technique to evaluate models is *k-*fold cross-validation. Cross-validation splits training data into *k* distinct splits; the model is trained on *k*-1 splits and evaluated on the split not used for training. The procedure is repeated *k* times ensuring that each split is used as a test set only once.	

**Fig. 2:**
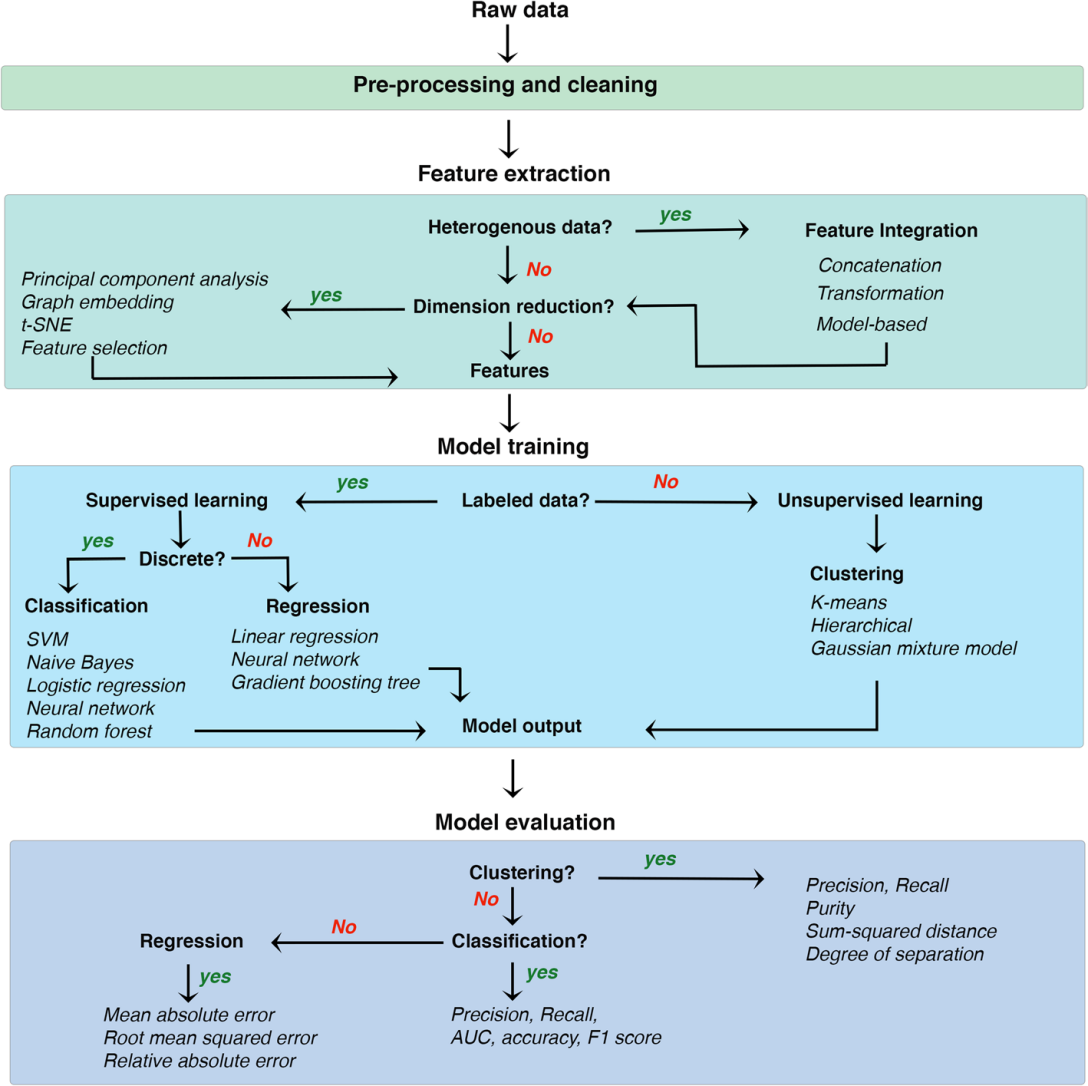
A typical machine learning workflow. While there are generally four main broad steps to develop a ML strategy (shaded boxes here and discussed comprehensively in the main text), a user is often left with multiple choices to select from a variety of tools/algorithms available for each step. The choice mainly depends on the dataset-specific factors and desired outcomes from the model. The workflow presented here can possibly guide decisions when choosing algorithms for developing and testing a data analysis strategy

On the technical level, ML approaches generally follow four logical steps (Fig. 2): (1) data pre-processing, (2) feature extraction, (3) model training, and (4) model evaluation. Data pre-processing is required to remove technical artifacts and noise from raw data obtained from various sequencing and imaging technologies. For example, in single-cell RNA-seq data, the majority of reported expression levels in scRNA-seq are zeros, which could be biologically driven (genes are not expressed in RNA at the time of sampling or may be silenced by epigenetic modifications), or technically driven (the expression level is not sufficient to be detected by sequencing technology), yielding many “dropouts”. Many imputation methods could be used to recover the gene expression loss due to dropouts, such as MAGIC [105], SAVER [106], scImpute [107], and RESCUE [108]. Another challenge of scRNA-seq is the batch effect. Due to the nature of some experimental scRNA-seq protocols, data are often captured and sequenced at different times, leading to confounding of biological variants. Few methods, including canonical correlation analysis (CCA) [85], mutual nearest neighbors [109], and Seurat [110], are commonly used to remove batch effects.

Several algorithms and statistical tests are frequently used to extract the most relevant features of a dataset, model training, and evaluation (Fig. 2). Most of the existing ML approaches can be categorized into three groups. Firstly, single-view learning involves algorithms applied to only single data types (either gene expression analysis or genotype inference or imaging analytics, etc.). In multi-view learning, machine learning algorithms are applied by integrating multiple sources of data. For example, understanding the combined effect of genotype and imaging together. We discuss below a few examples of single and multi-view learning applied to research on IDDs with some examples from other neurological and psychiatric disorders.

### Single-view learning

Single-view learning refers to using a single data type in an ML task. Below, we discussed some examples of how ML frameworks have been previously applied to single data types.

#### Neuroimaging data

The earliest applications of single-view models in neuroscience were applied to classify AD patients based only on image scans [111, 112]. In the aspects of neuroimaging data, Kim et al. [113] proposed a two-step deep neural network (DNN) on rs-fMRI data to classify schizophrenia patients from healthy controls and identify functional connectivity brain patterns that may be aberrant in SCZ. They first extracted functional connectivity measures using pairwise Pearson’s correlation analysis and then used these features as inputs to DNN for classification, achieving an error rate of 14.2%. Their proposed schemes and reported accuracy show improved ability to learn hidden patterns in brain imaging data, which may be useful for developing diagnostic tools for separate patient groups that in some cases are difficult to diagnose correctly due to overlapping symptoms such as psychotic bipolar and SZ patients [114]. Hazlett et al. [115] used a three-stage DNN to predict high-risk children diagnosed with autism at 24 months using surface area information from magnetic resonance imaging. Although the findings of their study do not have direct application to the larger population of children with ASD who are not known to be at high familial risk for ASD, they provide proof of principle that early brain changes occur during the period in which autistic behaviors are first emerging and demonstrate that early prodromal detection using a brain biomarker may be possible. Heinsfeld et al. [116] proposed an autoencoder-based DNN for ASD diagnosis using fMRI time-series data. The authors reported a classification accuracy of 70.0% for the entire ABIDE 1 dataset. The patterns of functional connectivity exhibits an anticorrelation of brain function between anterior and posterior areas, and this result corroborates current empirical evidence of anterior-posterior disruption in the brain connectivity in ASD. Li et al. [117] used a two-stage DNN approach where they first used a multi-layer convolutional neural network (CNN) to classify ASD samples from control samples using fMRI images, followed by prediction distribution analysis to detect saliency biomarkers, defined as important regions of interest (ROIs) in brain regions from the images that separated ASD from control individuals. These reliable ASD-associated biomarkers identified from accurate DNN classifiers are extremely helpful to understand the underlying roots of the disorder and can lead to earlier diagnosis and more targeted treatment.

Chen et al. [118] used EEG images and applied a CNN pipeline to classify children with ADHD and without ADHD with 90.29 ± 0.58% accuracy. Their proposed method could detect personalized EEG abnormalities for ADHD children at a precise spatial-frequency resolution. These personalized abnormalities detected could facilitate identifying potential neural pathways and planning treatments for children with ADHD. Tenev A et al. [119] introduced a method combining multiple SVM classifiers for classifying subtypes of classification of ADHD adults based on power spectra of different EEG measurement conditions. ASD-DiagNet used Auto Encoder (AE) as a feature extraction module. These features were fed to a perceptron for classifying ASD from healthy samples on the ABIDE 1 images, with an overall accuracy of 70.3% [120]. Their approach provides a method to leverage fMRI data, unlike the current psychiatric diagnostic process which is based purely on the behavioral observation of symptomology (DSM-5/ICD-10). Han et al. [121] used a three-stage deep learning approach to study the early onset of SCZ among 50 individuals (39 SCZ and 31 control) using rs-fMRI images, achieving an accuracy of 79.3% in classifying SCZ versus healthy individuals. The current diagnosis of SCZ is mainly relying on clinical manifestations by experienced clinicians because of the absence of stable and reliable biomarkers. Their results show that the functional connectivity at the resting state presented a good potential classification capacity to be a biomarker of clinical diagnosis.

#### Clinical data (EHR & behavioral data)

Recently, significant progress has been made in the clinical data acquisition methods and the storage infrastructure to build models using other data types. For example, a study scanned electronic healthcare records (EHR) of over 1 million patients in Wisconsin, USA, and developed an AI model to diagnose Fragile X syndrome [103]. In the preprocessing step, the researchers first mapped the diagnoses in the EHRs to the International Classification of Diseases (ICD) codes and worked with only those codes that appeared at least twice for a given individual. Then, they separated the EHRs into two classes following a supervised learning approach, one with FXS diagnosis and the other without FXS diagnosis. Finally, the model training step used the random forest (RF) classifier to find patterns in EHRs that characterize the FXS diagnosis. As a result, their models were able to identify FXS cases from controls with an approximately 80% accuracy and earlier than legacy methods in clinical diagnosis. Without relying on any genetic or familial data, this discovery-oriented study provides evidence of FXS association with a wide range of medical conditions including circulatory, endocrine, digestive, and genitourinary, in addition to mental and neurological disorders. Their approach could support healthcare provides to identify FXS patients and facilitate timely responses for their unique clinical needs in a multidisciplinary setting.

Single-view machine learning has also been applied to behavioral and electronic datatypes. For instance, Stahl et al. [122] applied optical flow and statistical pattern recognition to extract motion-based features from video recordings of young infants, then built an SVM-based early diagnosis assistance approach for cerebral palsy. The diagnosis of CP is often not set until the specific symptoms develop over the first year of life. The optical flow-based tracking method in this paper provides a powerful tool for a more automatic objective assessment system for early CP detection and for the first-trimester screening for Down syndrome based on prenatal clinical variables [123]. Koivu et al. [123] evaluated the usability and benefits of machine learning methods, such as SVM, neural network, for the first-trimester screening risk assessment of Down syndrome based on prenatal clinical variables, the best performing deep neural network model could achieve an AUC of 0.96. The deep learning architecture proposed in this paper performed comparatively to the current established prenatal screening software like LifeCycle™ (PerkinElmer, Waltman, MA, USA) which are based on the multivariate Gaussian risk calculation models, the screening accuracy would increase by adding more data for training, and lowering the overall costs of the screening program. Naderi et al. [124] used CNN-RNN architecture to diagnose SCZ, major depressive disorder, and BPD from speech audio recordings with an accuracy of 74.4%. Speech and language content are important aspects for the diagnosis and outcome prediction of mental illness. Due to the lacking standard of key linguistic elements for diagnosis and prognosis for clinicians to detect during mental state examination, the multimodal deep learning structure proposed in this paper would be a powerful tool to learn the effective representation of key audio and language characteristics for identifying mental disorders.

#### Multi-omics data

Single-view learning has been applied to identify genetic variants underlying a disorder and for prioritizing risk genes. For example, Cogill et al. obtained developmental transcriptomes from the BrainSpan Atlas to develop a model for classifying and prioritizing ASD risk genes [125]. They normalized the transcriptomes using standard methods and used gene expression values as features of known ASD genes to train SVM models, which achieved ~76% performance accuracy. Their proposed model did not need priori knowledge and could apply to long non-coding RNAs which implicated the etiology of ASD as well. Feng et al. converted the genotype intensity information into chromosome SNP maps and then applied 10-layer CNN to classify Down syndrome (DS) samples (63 DS, 315 control) [126]. They achieved a very high classification accuracy of 99.3%. The visualized feature maps and learned filters from their accurate classification model showed the local genomic patterns and correlated regional SNP variations in human DS genomes which provide opportunities to identify genomic markers for DS and insights for gene therapy. Zhou et al. [127] used the DeepSEA framework [127] to predict transcriptional and post-transcriptional effects among 1790 ASD simplex families. They studied the effects of noncoding mutations affecting RNA-binding proteins that enable identifying the functional impact of these previously orphaned noncoding mutations [128]. Liu et al. [129] applied CNN on SNP genotype data to classify 1033 ADHD and 950 healthy individuals. They achieved an accuracy of 90.18% and identified SNPs that helped classify ADHD and control individuals [129]. Their results indicated that their proposed deep learning model could capture the cumulative effect of insignificant SNPs and their contribution to ADHD, while GWAS failed. A similar strategy was used in the DeepAutism study, in which the authors collected SNPs from the SSC data from 2600 simplex families, used a chi-square test to select these variants, and applied CNN to classify children into ASD vs. healthy groups. Their classification achieved an accuracy of 88%, and they also identified genes having high contributory effects on ASD [130].

### Multi-view learning

Although much earlier work on ML applications focused on unimodal data (e.g., imaging, EHR, genomics) in isolation, the current trend has now shifted towards incorporating multimodal data [131–135]. Integration of data from multiple sources can provide complementary information about the system being studied and compensates for the shortcomings of missing or incomplete data from a single source. Multi-view learning typically follows these four logical steps outlined in Fig. 2: data cleaning and preprocessing, feature extraction, model training, and model evaluation. As outlined by Pillai et al. [136], the main goals of a data integration pipeline are to (1) reduce the dimensionality of individual views such that the most informative and complementary information is projected in the integrated/fused feature representation, (2) analyze the relationship between the views in order to gain insight on the relative contribution of each view to the learning task, and (3) handling missing data efficiently.

The integration of heterogeneous data is generally divided into two levels. The first level is a low-level data integration technique, often called “early fusion,” where features from different datasets are simply concatenated into a single-feature representation matrix before analysis. The second level of data fusion, referred to as “late fusion,” combines decision models trained on features of every individual dataset. In practice, early fusion can sometimes be troublesome if the different sources of datasets inherently possess many different types and representation forms (e.g., string, numeric, or graph). Zhang et al. proposed a unified way to integrate such feature representations that cannot be directly concatenated [137]. Their method constructs separate models for each dataset and then fuses them with a kernel combination technique. In addition, this method allowed them to provide individual weights to each view.

#### Integrating multimodal imaging data

While most studies included architectures dealing with a single-imaging data modality, newer studies are increasingly incorporating multimodal imaging analysis. For example, Colby et al. combined sMRI and fMRI and used a support vector machine (SVM) classifier to classify ADHD samples from the ADHD 200 dataset, albeit with a low accuracy of 55% [138]. Compared with the current diagnosis method in children by clinicians using subjective ADHD-specific behavioral instruments or by reports from the parents and teachers, machine learning tools using structural and functional magnetic resonance imaging data, and demographic information could a powerful tool to explore the abnormal brain circuitry in ADHD and to determine the underlying neural features related to ADHD. Libero et al. combined MRI, diffusion tensor imaging, and magnetic resonance spectroscopy and developed a decision-tree regression framework to identify differences in various clinical phenotypes (e.g., cortical thickness, neurochemical concentrations) among ASD individuals, with the classification accuracy of 91.9% [139]. Their results found alterations in cortical thickness, white matter (WM) connectivity, and neurochemical concentration for ASD individuals which would be used to explore the neural characteristics most relevant to ASD. Akhavan et al. [140] used a Deep Belief network on multimodal data (rs-fMRI, white matter, and gray matter and achieved an F1 score of 74% in classification of young age ASD (185 individuals, 116 ASD, and 69 control) by combining ABIDE I and ABIDE II datasets [140]. Prior to the eruption of reliable behavioral symptoms and untreatable complications, their deep learning model provided a powerful tool to extract the latent or abstract high-level features from rs-fMRI and sMRI for ASD diagnosis.

#### Integrating imaging and genomics data

Multiple studies focused on identifying gene-loci/SNPs that caused brain structural features (measured by sMRI) or functional connectivity (fMRI) changes, by integrating genetics with neuroimaging [141–144]. However, to understand the underlying molecular mechanisms resulting in the MRI measured brain structural/functional abnormality in IDDs, integrating transcriptome/epigenome with neuroimage data is necessary. Most of the previous research performed the studies by integrated analysis. For example, Berto et al. correlated the dynamic gene expression patterns with fMRI images to identify molecular mechanisms in memory encoding [145]. Zhao et al. developed a transcriptome-connectome correlation analysis method to integrate transcriptome data with fMRI connectome data and found age-specific cortex developmental gene signatures, which are highly associated with brain disorders [146]. Another study tried to understand human SCZ from an evolutionary perspective by comparing fMRI connectome between humans and chimpanzees. They found evolutionary genes expressing changes from transcriptome to support their findings in fMRI [147]. For deep canonically correlated sparse autoencoder (DCCSAE), the authors combined MRI scans and SNP genotype data to classify Schizophrenia [148]. DCCSAE contains two sparse stacked auto-encoders that learn non-linear relationships among SNPs and images and combine them using a fully connected network to classify schizophrenia and control samples. The joint analysis of fMRI and SNP data could extract significantly linked features that are highly correlated with SCZ and may get the insight into SCZ mechanism.

#### Integrating multi-omics data

Multi-omics data refer to the multiple “omes” of the biological system, such as genome, transcriptome, epigenome, proteome, methylome. Each type of genomic data yields specific biological knowledge and insights; integration of such data could illuminate non-linear relationships. For instance, in the context of Schizophrenia, Wang et al. recently integrated data from PsychENCODE, GTEx, ENCODE, CommonMind, Roadmap Epigenomics, and single-cell analyses into a deep-learning model based on gene regulatory networks and QTLs [149]. The interpretable deep-learning framework, the Deep Structured Phenotype Network (DSPN), captured relationships between genotype and phenotype by incorporating molecular phenotypes of genes (e.g., expression and chromatin state), predefined gene groupings (e.g., cell-type marker genes and gene co-expression modules), and traits (e.g., psychiatric disorders). The model showed a 6-fold improvement in trait prediction than traditional additive models [149]. Other frameworks, such as the Multi-Omics Factor Analysis (MOFA), can also capture the major source of variation in multi-omics datasets. MOFA decomposes individual modalities to provide a low-dimensional representation of the dataset and identifies shared and dataset-specific factors while handling missing data points [150]. To the best of our knowledge, multi-omics frameworks are yet to be applied to IDDs. Nevertheless, the technology is promising, and its application to IDD-related conditions is highly anticipated [151].

### Network biology

As discussed earlier, various genomic variants have been linked to IDDs. However, interpretation of such variants remains challenging, as genes involved in IDDs often converge into common cellular pathways. Studying functional relationships between genes could characterize pathways often associated with IDDs (e.g., neurogenesis, synaptic plasticity, and chromatin modification). Network biology is a powerful technique to study functional relationships between genes and identify modules with genes that act together and result in comparable phenotypes when mutated [152]. As such, network models can be thought of a feature extraction protocol from raw data. Network-based features of genes can be useful in a variety of applications from gene function prediction, gene prioritization, and drug repurposing, as discussed below.

#### Gene coexpression networks

Gene coexpression networks have been applied to study the human brain at bulk and single-cell levels [153, 154] and have various applications in neurological and neuropsychiatric conditions (reviewed in [155, 156]). For example, using 58 cortex samples and 21 cerebellum samples from cases with autism and controls, a study found a module (a group of coexpressed genes) enriched with known autism susceptibility genes [157]. The authors demonstrated the alterations in differential splicing associated with A2BP1/FOX1 levels in the ASD brain [157]. Gupta et al. [22] utilized region-matched autism and control brains to identify dysregulated neuronal and microglial genes in the cortical brain of autistic subjects. An analysis of 122 whole-hippocampus samples from patients with temporal lobe epilepsy found two modules conserved throughout the human cortex and in the mouse hippocampus [158]. These modules were found enriched for genetic variants associated with cognitive tasks and neuropsychiatric disorders [158]. GCN analysis has also revealed perturbations in Williams syndrome [159] and SCZ [160–162].

#### Integrative network models

Apart from transcriptome datasets, other data-types can also yield network-level information about cellular components. For example, a study recently integrated heterogeneous genomic data containing functional information (e.g., protein-protein, protein-DNA, protein-RNA, and metabolite-protein interaction data) from more than 14,000 publications [163]. The networks essentially represent functional relationship maps, which reveal tissue-specific functional roles of genes, tissue-specific rewiring of pathways, responses to perturbations, and relationships between diseases. The authors demonstrated that the resulting network models could serve as features in ML frameworks for gene prioritization. Another study by Wang et al. developed a technique called similarity network fusion (SNF) to integrate biological networks built from multiple resources (e.g., transcriptome, image, behavior) [164]. SNF has also been utilized to integrate epigenomic and transcriptome networks to reveal convergent molecular subtypes of ASD [21] and to define data-driven groups among children with ASD, ADHD, and OCD by integrating imaging and behavior measurements [165].

#### Network-based machine learning

Graph learning is one type of ML method specifically applied to learn patterns from biological networks. Traditional network analysis relies on heuristic features defined and engineered by humans (e.g., degree statistics, kernel function). Advanced machine learning methods (e.g., convolutional neural network) are generally designed for grid or sequence data; however, the information coded in biological networks is far more complex. To address this problem, transformation techniques based on deep learning, such as nonlinear dimension reduction (i.e., representation learning), have recently been implemented. Specifically, these transformation techniques embed network nodes into lower-dimensional spaces while maintaining the properties in the original space. In this manner, modern machine learning techniques can be directly applied within the lower-dimensional feature space. For example, Park et al. used manifold learning to analyze ASD patients’ MRI connectome and gene transcriptome, which pinpointed genes expressed in cortical/thalamic areas contributing to anomalies in brain circuits [166]. Another study from the same authors found an expansion of the structural network in the lower dimensional manifold space in adolescence, which was supported by gene enrichment results in their transcriptome analysis [167].

The other school of thought in the network-based ML domain argues that using the whole network, rather than a lower-dimensional representation, is generally more robust and accurate in classifying genes [168]. For example, Krishnan et al. [169] utilized the network connectivity profiles of known ASD genes in the human brain gene network to predict new genes potentially involved in ASD. Biological networks can also aid in the prioritization of hits in GWAS data [163, 170, 171] and drug repurposing [172]. For example, nominally significant *p*-values in GWAS outputs can be reprioritized by leveraging their connectivity patterns in a tissue-specific network [163]. The idea, aptly termed “NetWAS,” is to reprioritize hits that remain below typical user-defined subjective thresholds, but collectively may play important roles in pathways relevant to the GWAS.

#### Network medicine

Network medicine is an upcoming field that uses network biology methodologies to analyze drug-gene, drug-drug, and gene-gene interactions for the purposes of identifying existing drugs that can be repurposed for a particular disease [173]. Recent studies have shown the utility of network-medicine in drug repurposing for AD. For example, recently Xu et al. [174] leveraged single-cell RNA-seq, drug-target network, metabolite-enzyme associations, the human protein-protein interactome, and large-scale longitudinal patient data to identify gene network regulators in AD-associated microglia and astrocytes. Using these networks, the authors predicted repurposable drugs that are classified into various pharmacological categories, including anti-inflammatory, immunosuppressive, adrenergic beta receptor agonists, adrenergic alpha-antagonists, antihypertensive, and antineoplastic [174]. The authors validated their predictions using a large-scale, longitudinal patient database. Another recent study developed a disease module-based methodology for drug repurposing and implicated sildenafil as significantly associated with a decreased risk of AD [175]. Using neuron models derived from induced pluripotent stem cells, their study showed that sildenafil increases neurite growth and decreases phospho-tau expression [175], showcasing the benefits of in silico methodologies for generation of new testable hypothesis.

### Available AI and ML implementations

Various machine learning based tools have been applied in brain-related diseases and disorders, including general-purpose toolboxes (e.g., TensorFlow, Keras, PyTorch, and Scikit-learn) and toolboxes specifically designed for neurology purposes. For example, Abraham et al. have developed nilearn, which adapted scikit-learn into a higher-level machine learning toolbox for neuroimaging analysis [176]. Hahn et al. developed another Python library for neuroimaging-based machine learning, Brain Predictability toolbox, which incorporated standard machine learning prediction algorithms into a user-friendly platform for neuroimaging studies [177]. BrainSort is another ML toolkit specific for brain connectome data analysis and visualization [178].

Deep learning (DL) techniques are trendy in neurology due to the complexity of available data (i.e., high data volume, high dimensions, and incomplete data records). For example, DeepNeuron is a toolbox designed explicitly for neuron tracing [179], DeepBehavior is a DL toolbox for automated analysis of behavior data [180], and Braincode is a DL toolbox for EEG data decoding based Convolutional Neural Network (CNN) [181]. Recently, Lundervold et al. summarized state-of-the-art in CNN architectures [182]. In addition, these tools can benefit research on IDDs. For example, a recent Kaggle competition has built a machine learning pipeline pool for ASD prediction based on MRI measured morphological changes [183].

## Conclusions and future directions

Our review shows ML technologies could have a great potential for applications in IDD clinics for accurate early diagnosis as well as better understanding underlying molecular mechanisms. Speech, language, and behavior are important aspects of the mental illness diagnosis; however, due to the lacking standard for key linguistic elements for clinicians to diagnose, or a priori the eruption of reliable symptoms, the diagnosis mainly relies on the clinical manifestations by experienced clinicians. This made early diagnosis challenging before the untreatable complications. In these cases, machine learning could be a powerful early diagnosis assistance tool. For example, the diagnosis of cerebral palsy is often not made until a child is between one and 2 years old because specific symptoms of CP would usually develop over the first year of life. Stahl et al. [122] showed that video recordings of young infants to extract motion-based features to assist early CP detection. Akhavan et al. [140] combined rs-fMRI and sMRI to extract latent or abstract high-level image features to assist the early diagnosis in the cases of unclear behavioral symptoms for young children [140]. Naderi et al. [124] learned effective representation of key audio and language characteristics that could be helpful for the diagnosis of mental disorders. Overall, these studies show that data-driven machine learning tools could be an effective way to detect behavioral, linguistic patterns, and could assist the process of early diagnosis.

The complexity in overlapping genetic factors makes molecular stratification of IDD challenging. Nonetheless, the data and genetic knowledge generated from targeted sequencing of cohorts hold immense value in computational neurobiology. High confidence risk genes identified from genomic technologies can be utilized to create “gold standards” to benefit the development of evidence-based computational models of IDDs. Therefore, concerted efforts to extract such gold-standard information (e.g., pathogenic genes and variants) from the literature and documentation in centralized, open-access repositories are much desirable [184]. With the fast-increasing multi-modalities data types and number of datasets, machine learning has shown its potential to identify candidate biomarkers for further understanding the underlying mechanisms. Studies such as Liu et al. [129] and Cogill et al. [125] prioritized and explored the contribution and implication of insignificant SNPs neglected by GWAS, long non-coding RNAs to disease, which provide insights for understanding the complex etiology of disease. Many studies showed the integrated the transcriptome/epigenome with neuroimaging, and jointly analyzing multi-modalities to extract linked features that are highly correlated with the disease, may get insight into disease mechanism [145, 146].

We noted that ~27.4% of all IDD papers, we reviewed involved ASD, which highlights the relative lack of datasets specifically dedicated to other IDDs such as CP, FXS, and DS. We also noted that genomics and neuroimaging is the most widely studied modality in this context, with relatively fewer studies focusing on multi-modal data from the same cohort (Table 2). Importantly, our review highlights the potential applications of ML in diagnosis, biomarker discovery, and disease/patient classification within the context of various IDDs (Table 2). Several efforts are being made to generate large-scale multi-modal datasets for IDDs that should further accelerate widespread use of ML. For example, the Office of the Assistant Secretary for Planning and Evaluation’s Office of Behavioral Health, Disability, and Aging Policy (ASPE-BHDAP) is building a data infrastructure for publicly accessible state-level linked dataset pertaining to research on IDDs for 4 to 6 states. These data will link Intensity Scale, Medicaid claims, In-Person Survey, and other relevant data sources to aid in the evaluation of person-level predictors of outcomes prioritized by people with IDDs. Accumulation of such focused datasets, together with genome-level datasets, for example, from The London Down Syndrome Consortium (LonDownS), makes IDD ripe for ML-driven research. We anticipate that the existing ML-approaches tested on brain-disorders and other human diseases (e.g., cancer) will fuel predictive analysis in IDDs.

**Table 2:** Published research articles demonstrating machine learning applications to intellectual and developmental disabilities

Reference	Disorder *(in order of reference)*	Data type	Application
**Single-view learning**			
Koivu et al. (2018) [123]	Down’s Syndrome (DS)	NeuroImage	Disease risk assessment
Tenev et al. (2014) [119], Chen et al. (2019) [118], Eslami et al. (2019) [120]	ADHD, ADHD, ASD	NeuroImage	Patient classification
Wang & Avillach (2021) [130], Feng et al. (2018) [126], Liu et al. (2021) [129]	ASD, DS, ADHD	Multi-Omics, Behavior	Patient classification
Hazlett et al. (2017) [115], Heinsfeld et al. (2018) [116]	ASD	NeuroImage	Diagnosis
Heinsfeld et al. (2018) [116], Movaghar et al. (2021) [103]	ASD, FXS	Clinical	Diagnosis
Stahl et al. (2012) [122]	Cerebral Palsy (CP)	Behavior	Diagnosis
Ramaswami et al. (2020) [21]	ASD	Multi-Omics	Disease sub-typing
Voineagu (2011) [157], Johnson et al. (2016) [158], Gupta et al. (2014) [22]	ASD, Neurodevelopmental disease, ASD	Multi-Omics	Biomarker discovery
Liu et al. (2021) [129]	ADHD	Multi-Omics	Biomarker discovery
Cogill et al. (2016) [125]	ASD	Multi-Omics	Gene prioritization
Kimura et al. (2019) [159]	Williams syndrome	Multi-Omics	Cellular/molecular pathways
**Multi-view learning**			
Colby et al. (2012) [138], Libero et al. (2015) [139]	ADHD, ASD	NeuroImage	Patient classification
Jacobs et al. (2021) [165]	Multiple	NeuroImage, Behavior	Disease sub-typing

**Fig. 3:**
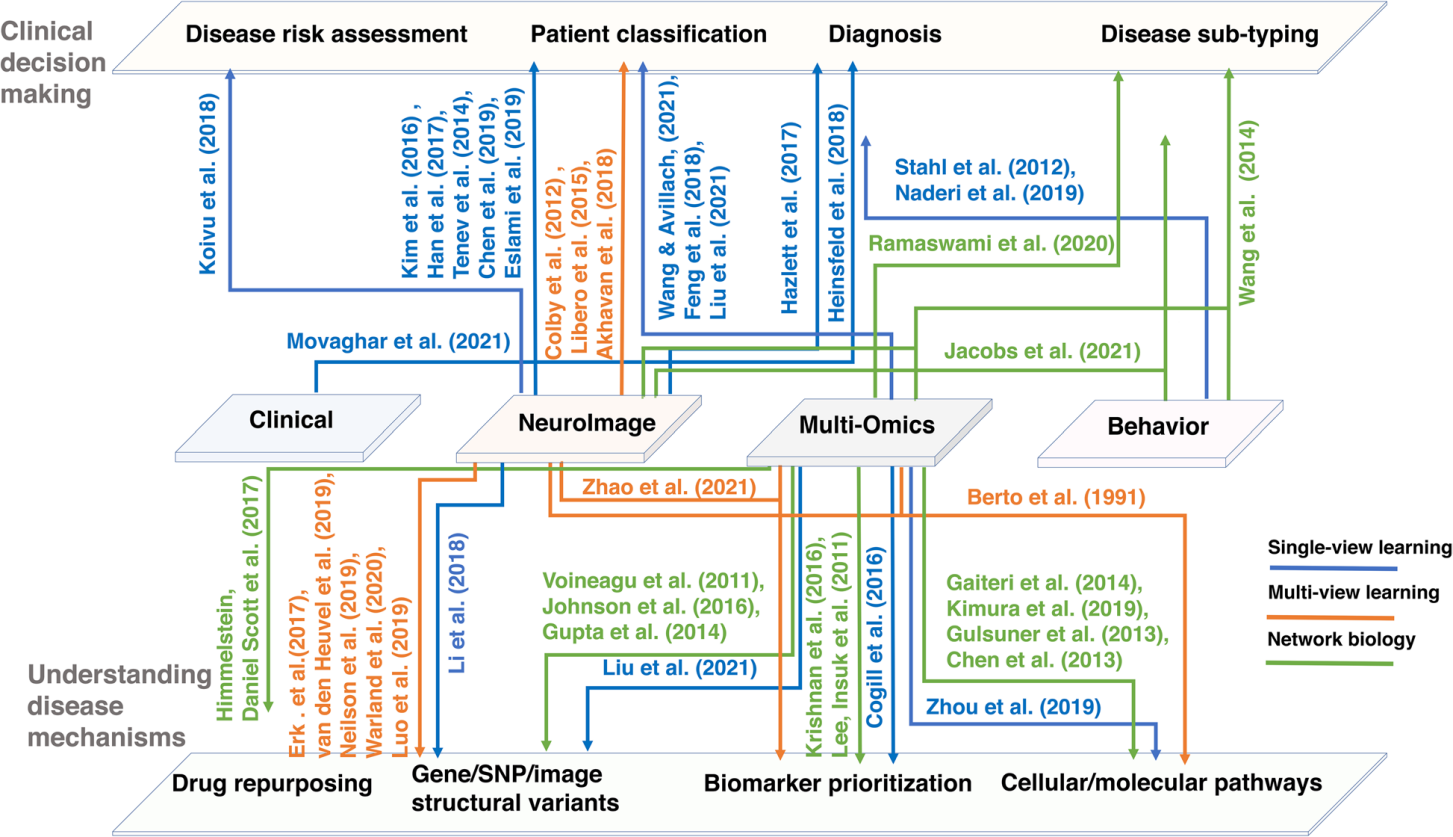
Applications of ML/AI-driven research. Some of the key studies we discussed in Machine learning methods and applications to IDDs section of this review are summarized. It is clear that the application of AI/ML (colored edges with references) to multimodal data types (depicted in the middle layer) has the potential to be useful in enhancing clinical decision-making (top layer) as well as developing a mechanistic understanding of IDDs (bottom layer)

The continuously advancing technologies for biological data acquisition, storage, and distribution have caught the attention of ML and AI experts. It is not surprising to see widespread applications of AI in understanding the complex nature of IDDs and other brain-related disorders (Fig. 3). Common comorbidities in children and adults within the IDD family of disorders are the features that make ML a suitable approach. For example, clustering analysis can help identify homogenous groups of people with similar comorbidities and disabilities. This has been explored, for instance, to identify groups of children with cerebral palsy and other similar comorbidities and disabilities in the National Survey of Children’s Health (NSCH). The study demonstrated that the groups defined by their ML approach is much better than those defined by traditional labels [185]. ML-based grouping has also been applied to find clinically meaningful strata within the spectrum of Tourette syndrome [186]. We anticipate that integrating multimodal data from large cohorts will help delineate overlapping factors and accurately characterize IDDs, ultimately improving our understanding of IDDs and leading to better clinical services in diagnosis and treatment/interventions. To that end, more research on comorbid conditions of IDDs, including sleep disorders, ADHD, depression, anxiety, and epilepsy, is needed before a data-driven precision-medicine framework is realized. As we are moving towards personalized medicine, we need ML systems to assist clinicians in making more accurate clinical decisions. It should be able to provide the decisions followed by proper reasoning and support. To this end, we can anticipate that the gap between the development of ML technology and clinical implementation will close quickly, if we continuously improve the resolution in datasets, computational capacity, proper evaluation metrics, and sophisticated interpretable algorithms. The community must address some key limitations/drawbacks for the successful application of ML approaches to IDD. We discuss some of these issues below.

It is important to note that processing large datasets using ML approaches can only tell what the diagnosis is. However, it is often desirable for clinicians to know why other possible diagnoses have been ruled out [185]. Furthermore, most of the existing IDD data is retrospective which means it’s historical data and many ML algorithms have been applied to such data but there is no guarantee that the same will perform on the real-world live data [187]. For example, even though it is easier to extract post-mortem brain data compared to live brain genomic data, it is required to curate a small set of such real-world data. In the other hand, missing data is another problem. It is difficult to get an ideal case scenario of truly complete data. So, ML algorithms should be able to handle such missing data in cases when it’s difficult to obtain complete data [188]. Furthermore, as the data is generated by multiple labs, these datasets come from different population with their own characteristics and distributions. Hence, the ML algorithms performing well on one data source might work poorly in another. This calls for guidelines to standardize heterogeneous datasets so that the ML algorithms are more generalizable.

While surveying the literature, we observed that many ML algorithms had been applied to brain imaging data more than other data modalities. While most of the studies involved single-view analysis and analyzed either imaging data or genetics data, relatively fewer studies utilized multimodal data integration approach but achieved only sub-par performance. A low performance could arise due to differences in data formats and dimensionalities. Future studies must focus on developing frameworks to efficiently integrate multimodal data with different structures. As deep learning models can learn complex representations of the data, fusing the representations of multimodal data can occur at multiple levels, and strategies for optimal fusing need to be researched and developed. Furthermore, most ML frameworks suffer from the problem of interpretability. The “black box” perception of ML has made it somewhat difficult to convince patients, clinicians, and regulators to unleash the potential of ML in clinics. We can anticipate that the gap between the development of ML technology and clinical implementation will close quickly, if we continuously improve the resolution in datasets, computational capacity, and sophisticated interpretable algorithms [189].

We also observed that most of the current studies have suffered from the curse of dimensionality (low sample size and high feature space). We believe that although the algorithms usually provide high levels of accuracy, the small sample size on which some of the models have been trained may not generalize well and have poor testing accuracy (i.e., low clinical usability). Other factors, such as class imbalance (for example, when the number of the disease samples are much lower than the healthy samples) can also confound the model. While reliable oversampling algorithms can be utilized to address such issues, other more sophisticated unbiased approaches can also be utilized. Furthermore, most ML frameworks suffer from the problem of interpretability and generalizability. Another problem is the performance metrics of these ML systems does not reflect clinical applicability. Most widely used metrics like ROC curves and accuracy metrics might not reflect in the clinical setting and often can be difficult to understand by the clinicians [187, 188]. For example, decision curve analysis (DCA) [190] can be used to improve upon the traditional model evaluation metrics (e.g., AUC) or other approaches that may require additional information on clinical consequences for individuals (e.g., financial costs, life-years lost, stress levels, treatment symptoms). DCA has been used in many different clinical evaluation applications [191].

Another time-sensitive issue, in our opinion, is accurate meta-data annotation and data sharing protocols. The different modalities of data are typically generated in different laboratories. For example, a genomics lab may not have access to imaging services and vice-versa. Therefore, centralized data storage infrastructures with open data could attract researchers from other fields where the real-world implementation of the ML technology is much more widespread (e.g., image recognition software). Furthermore, while current approaches focus on genomics, images, and clinical covariates, other behavioral data sources, like social media posts, health forum data, and text data from online support groups, can also be considered as data modalities that can provide complimentary information about individuals.

Finally, as privacy is a major concern in centralizing individuals’ data, informed consent, ethical considerations, and proper de-identification strategies to prevent potential misuse (e.g., model inversion and membership inference attacks) must be developed. Some methodologies, such as the Private Aggregation of Teacher Ensembles [192], attempt to provide a framework for using deep learning models on sensitive data (e.g., medical records), while maintaining a strong data privacy guarantee (that can ward off potential attacks from malicious users). The implementation of ML technology to enhance healthcare and digital medicine comes with its own set of ethical concerns [193–197]. AI and ML enthusiasts must consider suggestions and guidelines on transparency and reproducibility put forth by the expert scientific community [198–201].

## Data Availability

All results are provided in the manuscript.

## Abbreviations

ADHD: Attention deficit hyperactivity disorder
AE: Auto Encoder
AI: Artificial intelligence
ASD: Autism spectrum disorders
CNN: Convolutional neural network
CNV: Copy number variants
DCA: Decision Curve Analysis
DEG: Differentially expressed gene
DL: Deep learning
DNM: De novo mutations
DS: Down syndrome
EEG: Electro- encephalography
EHR: Electronic health record
EMR: Electronic medical record
ENCODE: Encyclopedia of DNA Elements
fMRI: Functional MRI
FXS: Fragile X syndrome
GCN: Gene coexpression network
GWAS: Genome-wide association studies
HITECH: The Health Information Technology for Economic and Clinical Health
IDD: Intellectual and Developmental Disabilities
iPSC: Induced pluripotent stem cell
LonDownS: London Down Syndrome Consortium
MEG: Magneto- encephalography
ML: Machine learning
MRI: Magnetic resonance imaging
NGS: Next-generation sequencing
PET: Positron emission tomography
rs-fMRI: Resting-state fMRI
sMRI: Structural MRI
SNF: Similarity Network Fusion
SNV: Single nucleotide variants
SV: Structural variations
